# Personality characteristics that are valued in teams: Not always “more is better”?

**DOI:** 10.1002/ijop.12511

**Published:** 2018-07-16

**Authors:** Petru Lucian Curşeu, Remus Ilies, Delia Vîrgă, Laurenţiu Maricuţoiu, Florin A. Sava

**Affiliations:** ^1^ Department of Psychology “Babeş‐Bolyai” University Cluj‐Napoca Romania; ^2^ Department of Organization Open University of the Netherlands Heerlen The Netherlands; ^3^ Department of Management and Organisation National University of Singapore Singapore; ^4^ Department of Psychology West University of Timişoara Timisoara Romania

**Keywords:** Big five‐factor model, Teamwork, Groups, Teams

## Abstract

This study investigates the relationships between personality traits and contributions to teamwork that are often assumed to be linear. We use a theory‐driven approach to propose that extraversion, agreeableness and conscientiousness have inverted U‐shaped relationships with contributions to teamwork. In a sample of 220 participants asked to perform a creative task in teams, we found that extraversion, agreeableness and conscientiousness were curvilinearly associated with peer‐rated contributions to teamwork in such a way that the associations were positive, with a decreasing slope, up to a peak, and then they became negative as personality scores further increased. We replicated the results concerning the non‐linear association between extraversion, conscientiousness and peer‐rated contributions to teamwork in a sample of 314 participants engaged in a collaborative learning exercise. Our results support recent claims and empirical evidence that explorations of personality–work‐related behaviours relationships should move beyond the linearity assumptions. We conclude by discussing the implications of our research for personnel selection.

Teamwork is both ubiquitous and important across a wide variety of organisational settings (Devine, Clayton, Philips, Dunford, & Melner, 1999) and teamwork skills have therefore emerged as valuable assets for personnel selection (Morgeson, Reider, & Campion, 2005). But what do teamwork skills entail? What makes one an effective and productive team member? Effective teamwork requires cooperation between team members, planning and coordination of individual actions as well as effective ways of dealing with interpersonal conflict (Baker & Salas, 1992). It follows that the individual knowledge, skills and abilities necessary to work effectively in teams cover two domains: the task domain (task specific knowledge and skills; e.g., skills for planning and task coordination) and the interpersonal domain (e.g., collaboration, communication and conflict resolution skills; Arthur et al., 2012; Baker & Salas, 1992; Stevens & Campion, 1994).

Based on a person‐environment fit framework (Caplan, 1987; Edwards, 2008; French, Caplan, & Harrison, 1982; Pervin, 1968) and, in particular, a situational congruence model—positing that individuals will perform better in environments that are congruent with their personalities (e.g., Pervin, 1968)—extraversion, conscientiousness and agreeableness are the personality dimensions that fit best the task and interpersonal demands associated with teamwork. In line with the “too much of a good thing” effect (Grant & Schwartz, 2011; Pierce & Aguinis, 2013) we extend the concept of fit and argue that effective contributions to teamwork are achieved at average rather than very high levels of conscientiousness, extraversion and agreeableness. In other words, personality dimensions previously acclaimed as catalysts for teamwork engagement (extraversion, conscientiousness and agreeableness) are only beneficial up to a point and when in excess, they have disruptive effects on contributions to teamwork. To substantiate this claim, we build on the distinction between desirable and undesirable personality traits (Coker, Samuel, & Widiger, 2002) to argue that due to the high incidence of undesirable traits at the very low and very high levels of extraversion, conscientiousness and agreeableness the best fit with teamwork is obtained at the average levels of these three personality dimensions. Therefore, the general aim of this study is to examine whether with respect to the personality dimensions mostly relevant to collaborative task accomplishment (extraversion, agreeableness and conscientiousness), there is some optimal level that ensures a good person‐team environment fit. We also aim to replicate these non‐linear associations between personality and contributions to teamwork in different groups operating in two cultural contexts.

## Theory and hypotheses

Contributions to teamwork are often described along two dimensions: contributions to the task and contributions to the interpersonal atmosphere, a distinction that also fits the task and social roles enacted by team members (LePine, Buckman, Crawford, & Methot, 2011). The high degree of interdependence associated with collaborative team tasks makes contribution to the team task and team atmosphere intrinsically linked and previous research reports correlations ranging between .77 and .85 for the individual contributions to the task and their involvement in interpersonal interactions (Loughry, Ohland, & Moore, 2007; Ohland et al., 2012). Given the social nature of collaborative tasks and the high correlations reported in the literature between task contributions and contributions to interpersonal interactions, we will further on, jointly, refer to these two dimensions as contributions to teamwork. In line with Sonnentag and Volmer (2009), we argue that individual contributions to teamwork are observable behaviours in the task and interpersonal domains, and we use a Situational Judgement Test approach (Lievens & Sackett, 2012) to collect peer ratings of one's contributions to teamwork in order to evaluate his/her teamwork skills.

Although research to date extensively tested the effects of personality dimensions on team performance (Bell, 2007; Peeters, Van Tuijl, Rutte, & Reymen, 2006) and explored the association between personality and teamwork quality, most research claimed (Driskell, Goodwin, Salas, & O'Shea, 2006) or attempted to find (Morgeson et al., 2005) linear associations between personality dimensions and various facets of effective teamwork. So far, convergent results in the literature point towards the beneficial role of three personality dimensions (extraversion, conscientiousness and agreeableness) for teamwork and collaborative task performance (Driskell et al., 2006; Peeters et al., 2006; Van Vianen & De Dreu, 2001). Aligned with these results and theoretical claims, we expect that extrovert, conscientious and aggregable group members will contribute more effectively to teamwork as compared to members scoring low on these personality dimensions.

The “too much of a good thing” (TMGT) meta‐theoretical framework describes situations in which “ordinarily beneficial antecedents (i.e., predictor variables) reach inflection points after which their relations with desired outcomes (i.e., criterion variables) cease to be linear and positive” (Pierce & Aguinis, 2013, p. 315). The TMGT effect has the potential to explain a substantial number of non‐linear relationships between variables explored in management research (Pierce & Aguinis, 2013) and psychology (Grant & Schwartz, 2011). In the broader literature on personality and general work‐related outcomes, recent conceptual developments and empirical work (Borkenau, Zaltauskas, & Leising, 2009; Bozionelos, Bozionelos, Polychroniou, & Kostopoulos, 2014; Carter et al., 2014; Le et al., 2011; McCord, Joseph, & Grijalva, 2014) have reconsidered the linearity of the links between the personality dimensions and work‐related behaviours.

Borkenau et al. (2009) showed that relations between trait level reflected by self‐reports and trait level desirability as evaluated by peers comprise curvilinear components for 30 items measuring Big Five (Borkenau et al., 2009). Moreover, Le et al. (2011) showed that the association between personality dimensions (e.g., conscientiousness, extraversion) and job‐related behaviours and outcomes is positive up to a point (the peak of an inverted U‐shaped curve) and then the association is less strong, or it becomes negative. The specific aim of this study is to extend these insights to teamwork skills and test the curvilinear associations between personality traits and peer‐rated contributions to teamwork.

The use of various person‐environment theories and models in organisational psychology has been a prolific endeavour over the past few decades, and this research has examined a variety of types of fit as well as many types of outcomes (see, e.g., Edwards, 2008). What we are concerned with, in this study, is a type of abilities‐demands fit predicting that individuals with certain personalities function more effectively in teamwork environments. Whereas this general prediction is neither new nor surprising, we depart from previous research by specifically proposing that there exist optimal levels of personality traits where fit and, therefore, effective teamwork, is best. In other terms, we propose that personality traits known to be associated *linearly* with effective contributions to teamwork (e.g., extraversion; Barrick, Stewart, Neubert, & Mount, 1998) actually have a *curvilinear* relationship with effective contributions to teamwork because fit with the teamwork environment does not imply maximising certain personality characteristics but rather an optimal level of those personal characteristics.

From the Big Five Model, three personality dimensions have both task and interpersonal (positive) correlates that make them relevant for teamwork, namely extraversion, conscientiousness and agreeableness. As collaborative tasks are social in nature, agreeableness and extraversion, as interpersonal traits and conscientiousness as task‐related trait are expected to influence effective contributions to teamwork through interpersonal interactions, cooperative groups norms and task engagement (Barrick et al., 1998; Carter et al., 2014; Gonzalez‐Mulé, DeGeest, McCormick, Seong, & Brown, 2014). We use the TMGT effect (Grant & Schwartz, 2011; Pierce & Aguinis, 2013) to argue that these three positive personality dimensions (extraversion, conscientiousness and agreeableness), when in excess disrupt contributions to teamwork. In line with the person‐environment fit theories (Edwards, 2008; French et al., 1982; Pervin, 1968), we posit that the some of the maladaptive traits subsumed to extremely high or low scores on extraversion, conscientiousness and agreeableness reflect a misfit between personality and the teamwork requirements. In Table 1 we summarise some of the maladaptive teamwork‐relevant traits mentioned in the personality literature as associated with high and low levels of extraversion, conscientiousness and agreeableness.

**Table 1:** Teamwork‐relevant maladaptive personality traits at the extremes of conscientiousness, extraversion and agreeableness

	Task contribution		Contribution to interactions	
Personality dimension/Score	High personality score	Low personality score	High personality score	Low personality score
C	Perfectionist Inflexible Obsessive	Disorderly Careless Wasteful	Leisureless Defensive Hypersensitive Moody	Neglectful Uncontrolled
E	Superficial Dominant Exaggerative Egoistic	Withdrawn Reclusive Detached (from the task)	Flaunty Showy Overly reactive	Unfriendly Distant Solitary
A	Lenient Ingratiating Submissive	Harsh Competitive Unwitting	Deceivable Dependent Gullible	Deceitful Heartless Treacherous

*Note*: The table integrates teamwork‐relevant maladaptive traits mentioned by Coker, Samuel, & Widiger (2002), McCord et al. (2014) and Carter et al. (2014).

With respect to specific personality dimensions, Barrick et al. (1998) identified extraversion as the most consistent personality predictor of positive interpersonal interactions in teams. In line with person‐environment fit models, we argue that extraverted individuals fit well with work situations that require interpersonal interactions. On the contrary, introverts are reserved, less willing to engage in social interactions and to contribute to group debates (Mohammed & Angell, 2003), and thus they are likely to be perceived as not contributing effectively to teamwork. Extraverted individuals, on the other hand, tend to be energised by working in a team that facilitates cohesion and is ultimately perceived as beneficial for teamwork processes (Van Vianen & De Dreu, 2001). Although in general extraverts are perceived to have good interpersonal skills because they are naturally talkative, enthusiastic, sociable, warm, trusting and fun‐loving (Costa & McCrae, 1992), extreme extraverts may disrupt interpersonal relations as they are ostentatious and showy, superficial and overly reactive (Coker et al., 2002).

Due to their proclivity for social situations, extraverts are likely to be active during group meetings and actively engage in group debates. Therefore, extraversion is expected to be, up to a point, positively associated with effective teamwork skills. However, people with extremely high levels of extraversion may lack listening skills and may be perceived as talking too much (Costa & McCrae, 1992), have a tendency to dominate conversations and although they may become the formal or informal leader in the work team (Peeters et al., 2006), they may be perceived as not contributing effectively to teamwork. Broadly speaking, those who are very extraverted enjoy highly visible team (leading) roles (Lord, 2007). Therefore, others may perceive them as being dominant leaders (Ames & Flynn, 2007) rather than “good team players.” As team members, extreme extraverts may engage in excessive socialisation, unrelated to the task, dominate the discussions with their views, exaggerate their individual skills and contributions and as such distract the team from its collective goals (Murphy, 1996). In the same vein, Barry and Stewart (1997) showed that teams composed of too many extraverts lacked task focus and clear goal orientation. More specifically, they identified an inverted U‐shaped relationship between the number of extraverts in the team and general team effectiveness. Therefore, both theory and empirical evidence point towards a curvilinear association between extraversion and contributions to teamwork; that is, extraversion should be positively associated with contributions to teamwork up to a point and then the association would become negative. We therefore hypothesize that:

H1. There will be an inverted U‐shaped relationship between extraversion and effective contributions to teamwork.

Agreeableness is a personality dimension that captures attributes closely associated with interpersonal relations. Agreeableness is particularly relevant for teamwork as it is a core dimension for person‐environment fit especially in situations involving interpersonal conflict (Ilies, Johnson, Judge, & Keeney, 2011). People scoring high on agreeableness are described as cooperative, considerate, trusting, easy going, empathic, friendly, supportive and receptive to the perspectives of others (Costa & McCrae, 1992). Perhaps more than any other dimension of the Big Five Model, agreeableness involves socially valued personal attributes (Graziano & Tobin, 2002). Therefore, it contributes to the fit between the person and situational requirements associated with teamwork. As agreeableness is associated with the willingness to cooperate and good conflict management skills, it is likely that, up to a point at least, agreeableness is positively related to effective contributions to teamwork.

However, group members with extremely high scores on agreeableness may tend to prioritise the needs of colleagues over their own needs or the broader needs of the team (Lord, 2007). The downside of such tendencies is that they do not support independent or critical thinking, and can be considered by other members of the as team dependent or unprincipled (because of a yielding position) and, as a consequence, not contributing effectively to teamwork. The tendency to please others may results in overlooking the errors made by other teammates, not disclosing performance deficits in order to avoid conflict and withholding dissentful opinions in order to be ingratiating, all detrimental to collaborative task performance (McCord et al., 2014). Also in terms of collective task engagement, extreme agreeable team members may take over the tasks of their underperforming teammates or avoid to refuse excessive tasks and ultimately due to individual task‐overload fail to perform and contribute to the collective goal accomplishment (Murphy, 1996). Highly agreeable individuals may come across to others as naive, submissive, conflict‐averse and non‐competitive (Graziano & Tobin, 2002; Howard & Howard, 2001), while group members scoring low on agreeableness may be perceived as unfriendly and untrustworthy. Thus, agreeableness is expected to be positively associated with contributions to teamwork up to a point, and then the association is expected to become negative. We, therefore, hypothesize that:

H2. There will be an inverted U‐shaped relationship between agreeableness and effective contributions to teamwork.

Conscientiousness has been shown to be the most consistent predictor of performance across jobs (Barrick & Mount, 1991) and is a strong predictor of individual‐based performance because of the high achievement motivation of conscientious individuals (e.g., Richardson & Abraham, 2009). As task achievement is a core element of teamwork, it follows that conscientiousness is highly relevant for the fit between the person and the task dimension of teamwork. Individuals scoring high on conscientiousness are responsible, controlled, orderly, cautious, meticulous, and they have a strong will to achieve difficult goals (Costa & McCrae, 1992). Due to the high task focus, conscientiousness is expected to be positively associated with contributions to collaborative task accomplishment as conscientious members are likely to be highly committed to the group task (Peeters et al., 2006). In addition, in small group settings, conscientiousness is also likely to be beneficial for the emergence of interpersonal trust and cooperation, as conscientious persons are perceived to be reliable and trustworthy (O'Neill & Allen, 2011).

However, people scoring very high on conscientiousness are often perfectionists, overly focused on their personal goals, and so concerned with flawless task execution that they may delay the achievement of collective goals; they will not engage in organisational citizenship behaviours, leading to relationship tensions and eventually preventing the team from achieving its collective goals (Carter et al., 2014). Highly conscientious group members also tend to react to negative feedback and critical work events by engaging in counterproductive work behaviours (Carter et al., 2014), that will eventually be perceived by others as detrimental to teamwork. Moreover, as team debates often revolve around relational topics, which are not related to accomplishing task goals, people scoring extremely high on conscientiousness may discourage such debates because such individuals have a strong task orientation and are overly concerned with the goal achievement. Recent work on conscientiousness and work‐related behaviours suggests that beyond a certain point conscientiousness may no longer be positively related to task performance (Le et al., 2011). In small groups settings, people scoring very low on conscientiousness are likely to engage in social loafing (Mohammed & Angell, 2003), while the ones scoring very high are likely to be perceived as overly demanding, compulsively focused on their task and/or stubborn (Le et al., 2011), therefore, both the ones scoring very low or very high are likely to be perceived as not contributing effectively to teamwork.

H3. There will be an inverted U‐shaped relationship between conscientiousness and effective contributions to teamwork.

Although research to date suggests that openness to experience could also be non‐linearly related to job outcomes (Bozionelos et al., 2014; Vasilopoulos, Cucina, & Hunter, 2007) and such non‐linearity could be theoretically grounded (McCord et al., 2014) we consider the empirical evidence insufficient in order to hypothesize that openness to experience has a non‐linear relation with contributions to teamwork. Neuroticism, on the other hand, is a global indicator of maladaptive functioning (Claridge & Davis, 2001), and was explored as a predictor of organisational citizenship behaviours or counterproductive work behaviours (Duffy, Shaw, Scott, & Tepper, 2006; Le et al., 2011; Ohana, 2016). Neurotic individuals are less central in the advice and friendship networks in teams (Fang et al., 2015; Klein, Lim, Saltz, & Mayer, 2004) and often generate negative interpersonal dynamics and negative affectivity in teams (LePine et al., 2011), therefore it is unlikely that neuroticism has a non‐linear association with contributions to teamwork. Nevertheless, given its strong relation to negative affectivity, neuroticism should be accounted for when testing the non‐linear associations of the other personality traits with contributions to teamwork in order to disentangle the plausible effect of the maladaptive traits at the poles of the other personality dimensions from the generic maladaptive traits subsumed to neuroticism (Carter et al., 2014). Carter et al. (2014) also suggested that the five personality dimensions should be considered together when estimating such non‐linear patterns. Therefore, we will also explore the non‐linear association between neuroticism and openness to experience on the one hand and contributions to teamwork on the other hand.

## METHOD

### Samples

This study used two independent samples, the first comprised 220 students (83% women, with an average age of 20.67 years old) enrolled at a large public university in Romania (further on labelled as the Romanian sample) and the second comprised 314 students (45.5% women with an average age of 19 years old) enrolled at a Dutch university (further on labelled as the Dutch sample). The student groups in the two samples were engaged in qualitatively different tasks. The groups in the Romanian sample were *ad‐hoc* groups (having 3–6 members with an average group size of 4.56) asked to perform a single task in laboratory setting, while the groups in the Dutch sample (having 3–7 members with an average group size of 5.32) were established groups (had to work together for the whole semester on various tasks during and outside class). Likewise, while the composition of the Romanian groups has been imposed by researchers, the Dutch participants were allowed to decide themselves the composition of their groups (they were allowed to select their teammates). Therefore, using the two different types of samples allows us to test the extent to which our hypotheses are generalizable across different cultural contexts and different types of group tasks.

### Procedure and measures for the Romanian sample

Participants in the Romanian sample were asked to participate in a creative group exercise after signing an informed consent form. They were first asked to fill out a personality inventory and then asked to solve the creative task (e.g., find a way to release an egg into a bowl, such that the egg will not break) in teams having three to six members. Each group received several objects they could use during the task: six drinking straws of equal sizes, a 1‐m long plastic stripe, duct tape and a plastic bowl. The task was collaborative in nature, all members had similar roles and were asked to collaborate in solving the task. After finishing the task, the participants were asked to fill out a questionnaire with a scale for self‐reported teamwork skills and to evaluate the contribution to teamwork for each of their teammates.

The Big Five personality dimensions were evaluated with NEO Five‐Factor Inventory (NEO‐FFI; Costa & McCrae, 1992). This is a questionnaire that assesses each factor with 12 items. For each item, participants have to indicate the extent of agreement with ratings in a 5‐point Likert format. We used the Romanian version of the NEO‐FFI by Iliescu, Minulescu, Nedelcea, and Ispas (2009) that reported a satisfactory level of internal consistency.

Peer‐rated contributions to teamwork were evaluated by asking each group member to evaluate the extent to which each of their team mates contributed to the task (“Please rate the contribution of each team member to the task:” 1 = *unsatisfactory* to 10 = *substantial*) and the group atmosphere (“Please rate the contribution of each team member to the work atmosphere within the group:” 1 = *unsatisfactory* to 10 = *substantial*) while performing the task. Because the two scores were highly correlated (.85), a result similar with correlations previously reported in the literature (Loughry et al., 2007; Ohland et al., 2012), they were averaged to obtain a composite score for contributions to teamwork as evaluated by the peers. Thus, the effective contribution to teamwork score covers both domains: task participation and interpersonal orientation. Because each team member was evaluated by several peers, we computed the within‐group agreement index (the Rwg coefficient presented in James, Demaree, & Wolf, 1984) for the contributions to teamwork score. The Rwg ranges from .75 to 1.00, with an average of .83 and thus indicates an acceptable level of rater agreement (Bliese, 2000) and the scores were averaged across raters for each participant.

In order to test the convergent validity, a measure of self‐reported teamwork skills was also used. The scale had three items (“I have very good teamwork skills,” “I am able and willing to work together with others in a team,” and “I have a real talent in collaborating together with others in a team”) and the answers were recorded on a 5‐point Likert scale ranging from 1 = *strongly disagree* to 5 = *strongly agree*). Cronbach's α for this scale was .79. The two evaluations of teamwork skills were significantly correlated (.49 when correcting for the unreliability of the scales), supporting the validity of the peer‐rated scores based on the observed “in role” behaviour, in the team.

Team member familiarity was evaluated using a similar item format as the peer‐rated contributions to teamwork. The respondents were asked to answer the following statement: “Please rate the extent to which you were familiar with each of your teammates before you started the task:” 1 = *not familiar at all* to 10 = *very familiar*). The evaluations were averaged across raters for each team member.

### Procedure and measures for the Dutch sample

The participants in the Dutch sample were enrolled in a course that used collaborative learning groups, and they completed the questionnaires as part of their regular course activities. In the first workshop, the participants were asked to fill out a personality inventory (they were asked to interpret their personality profile as part of an individual assignment) and they were asked to form small groups (having 3–7 members) and participate in a collaborative learning exercise (they were asked to build a cognitive map with a number of course‐related concepts provided by the lecturer; for more details on the procedure see Curşeu & Pluut, 2013). Students received 20 course‐related concepts and were asked to collectively organise them in a way that reflected their group understanding of the course domain. The exercise lasted for 45–50 minutes and in general students were very engaged in the task as the task required intense debates on the relation and organisation the concepts and lots of coordination efforts for the positioning of the concepts on the cognitive map. At the end of the exercise, the students were asked to present their cognitive map in front of the class and were then asked to fill out the scale for self‐reported teamwork skills and to evaluate the contribution to teamwork for each of their teammates.

The Big Five personality dimensions were evaluated using a short version of the Big Five Inventory (Rammstedt & John, 2007). The questionnaire contains two items for each of the big five dimensions and although, similar to previous samples (Credé, Harms, Niehorster, & Gaye‐Valentine, 2012) the Cronbach's α for our sample are rather low (*E* = .52; *A* = .48; *C* = .60; *N* = .69; *O* = .46) previous research showed good validity and test–retest reliability for the short personality measure (Rammstedt & John, 2007). The peer‐rated contributions to teamwork and team member familiarity were evaluated using the same items as for the Romanian sample rated on a 5‐point Likert scale (1–5). Similar to the Romanian sample, the two indicators of contributions to teamwork were highly correlated (.77), and we summed the score to obtain a global indicator of contributions to teamwork. The within‐group agreement index ranged from .75 to 1.00 with an average of .84, indicating good inter‐rater agreement for each of the participants. Therefore, we averaged the scores across raters for each participant. Finally, the self‐rated teamwork skills were evaluated using the same three items as for the Romanian sample, and the Cronbach's α for this scale was .75. The self‐rated teamwork skills are positively and significantly correlated with the composite variable of peer‐rated contributions to teamwork (correlation corrected for attenuation is .40).

## RESULTS

Means, standard deviation and correlations for the variables considered in the study are presented in Table 2 (intercorrelations for the Romanian sample are presented below and the ones for the Dutch sample above the diagonal). As indicated by the score range of the personality dimensions, range restriction (Pierce & Aguinis, 2013) is not likely to be a problem in our sample when testing the curvilinear relations between personality dimensions and the effective contributions to teamwork.

**Table 2:** Means standard deviations and correlations

	Mean	*SD*	Range	1	2	3	4	5	6	7	8	Range	Mean	*SD*
1. Neuroticism	1.90	0.63	(0.42‐4)	1	−.16**	−.07	−.12*	−.03	−.18**	−.09	−.00	(1;7)	3.14	1.30
2.Extraversion	2.49	0.47	(1‐4)	−.48**	1	.05	.07	.18**	.21**	.23**	−.01	(1.5;7)	5.09	1.04
3.Openness to experience	2.42	0.47	(1.25‐3.67)	−.10	.14*	1	.00	.17**	.00	.04	−.13*	(1;7)	4.42	1.14
4.Agreeableness	2.59	0.45	(1.25‐3.67)	−.18**	.33**	−.03	1	.20**	.17**	.23**	−.01	(1.5;7)	5.07	.99
5.Conscientiousness	2.71	0.58	(0.83‐3.83)	−.27**	.34**	.02	.21**	1	.15**	.21**	−.04	(2;7)	4.85	1.15
6.TWK skills (self report)	3.75	.67	(1.33‐5)	−.19**	.34**	−.03	.12	.29**	1	.30**	.00	(2;5)	3.73	.54
7.Contributions to TWK	8.38	1.18	(2.5‐10)	−.12	.23**	−.16*	.06	.21**	.35**	1	.10*	(2.67;10)	7.98	1.29
8. TM familiarity	5.36	2.10	(1‐9.25)	.02	.01	−.03	−.06	.03	.07	.39**	1	(1;5)	2.16	.80

*Note*: Romanian sample (*N* = 220) correlations are given below the diagonal, means, SDs and range are presented in the first three columns; Dutch sample (*N* = 314) correlations are given above the diagonal, means, SD and range are presented in the last three columns. TM = team member; TWK = teamwork.

* *p* < .05.

** *p* < .01.

^***^ *p* < .001.

Because individual group members (level 1) are nested in groups (level 2), evaluations of contributions to teamwork might be influenced by various group level dynamics (e.g., within‐group conflict). Therefore, in order to account for the non‐independent nature of the data, we used a multilevel modelling approach to analyse the data. Although all variables of interest are measured at the individual level (level1), by using multilevel modelling analyses we accounted for the variation of the intercept from one group to another (in random‐intercept models) or the variation of the slopes from one group to another (in random‐slopes models).

Following the recommendations provided by McCoach (2010), we built the multilevel models sequentially, and we estimated four multilevel models for each sample. For both samples, the statistical results for each model are presented in Table 3. First, we estimated unconditional (or *null*) models to investigate the proportion of variance that can be accounted by each level. On the Romanian sample, 48.23% of the criterion variance is at level 1, while on the Dutch sample 19.48% of the variance is within groups.

**Table 3:** Fixed effects estimates (top) and variance–covariance estimates (bottom) for models of the predictors of peer‐rated team work skills

Parameter	Model 1		Model 2		Model 3		Model 4			
Fixed effects										
	NL	RO	NL	RO	NL	RO	NL		RO	
Int.	7.99* (.10)	8.42* (.13)	7.98* (.07)	8.58* (.20)	7.98* (.12)	8.65* (.19)	7.98* (.11)		8.55* (.18)	
Level 1										
Gender			−.05 (.15)	.19 (.20)	−.02 (.14)	.27 (.19)	−.05 (.13)		.16 (.16)	
Fam.			.17 (.09)	.17* (.04)	.23* (.10)	.15*(.03)	.29* (.09)		.16* (.04)	
N			−.04 (.06)	−.06 (.11)	−.04 (.06)	−.06 (.11)	−.04 (.06)		−.10 (.13)	
E			.24* (.07)	.34* (.16)	.17*(.07)	.31* (.14)	.19* (.06)		.27* (.13)	
O			.02 (.06)	−.20 (.14)	.02 (.06)	.20 (14)	.01 (.06)		−.15 (.12)	
A			.24* (.07)	.08 (.14)	.13† (.07)	.08 (.14)	.09 (.07)		−.05 (.13)	
C			.16* (.06)	.24* (.11)	.12* (.06)	.08 (.12)	.14* (.06)		.03 (.11)	
N^2^					.01 (.03)	.08 (.11)	.01 (.02)		−.07 (.12)	
E^2^					−.14* (.05)	−.40* (.20)	−.14* (.04)		−.38* (.18)	
O^2^					.01 (.04)	−.47* (.21)	.01 (.05)		−.41* (.20)	
A^2^					.09 † (.04)	−.56* (.20)	−.04 (.06)		−.41* (.18)	
C^2^					−.16* (.04)	−.35* (.12)	−.15* (.04)		−.32* (.11)	
Random parameters										
Level 2										
Int./Int. (σ^2^_a0_)	.34*	.68*	.34*	.48*	.34*	.45*		.32*		.41*
							O/O (σ^2^_a1_)	.03*	Fam./Fam (σ^2^_a1_)	.04†
							A^2^/A^2^ (σ^2^_a2_)	.07*	N/N (σ^2^_a2_)	.30*
							O^2^ /O^2^(σ^2^_a3_)	.05*	Fam/Int. (σ^2^_a10_)	−.03*
							O/Int (σ^2^_a10_)	−.01	N/Int. (σ^2^_a20_)	.18*
							A^2^/Int. (σ^2^_a20_)	−.06*	Fam/N (σ^2^_a12_)	−.01
							O^2^ /Int. (σ^2^_a30_)	−.02		
							O/A^2^ (σ^2^_a12_)	.01		
							O/O^2^ (σ^2^_a13_)	.04*		
							A^2^/O^2^ (σ^2^_a13_)	−.01		
Deviance	1031.91	633.64	1017.15	582.64	1006.73	563.41		977.48		544.26

*Note*: A = agreeableness; C = conscientiousness; E = extraversion; Int. = intercept; N = neuroticism; NL = results for the Dutch sample; O = openness to experience; RO = results for the Romanian sample; TM Fam = team member familiarity.

† < .10.

* *p* < .05.

Second, we entered gender, familiarity among group members, and the grand mean centered scores for the five personality dimensions as linear effects (Model 2). Familiarity among the group members was included as a control variable because it influence peer ratings as illustrated by the fact that it was positively associated with the peer‐rated contributions to teamwork (γ_20_ = .16, *SE* = .04, *p* < .01 for the Romanian sample, γ_20_ = .29, *SE* = .09, *p* < .01, for the Dutch sample). Moreover, familiarity it is also likely to co‐vary with personality as similarity in personality profiles is an important factor in pre‐established friendship ties. Gender was entered as a control variable as according to the social role theory (Eagly, Wood, & Diekman, 2000) and recent empirical evidence (Curşeu, Pluut, Boroş, & Meslec, 2015; Woolley, Chabris, Pentland, Hashmi, & Malone, 2010) men and women differ in their communal (teamwork oriented) and agentic (task oriented) traits and their contribution to teamwork might differ. In order to allow for an easier interpretation, all predictors were centered on the sample mean (grand mean centering). In the third step the quadratic terms were added (Model 3). As Models 2 and 3 were random‐intercept models (we controlled for the random variation of the intercept from one group to another), in the next step we investigated whether the slopes varied randomly from one group to another. When building the random‐intercept–random slopes models (Model 4), we first investigated whether the variation of each slope was statistically significant. Next, we specified a model with all the random slopes that had significant variance in the previous stage. Finally, we eliminated the random slopes that became statistically non‐significant, as a result of their simultaneous estimation. The results of the four models are presented in Table 3.

**Figure 1:**
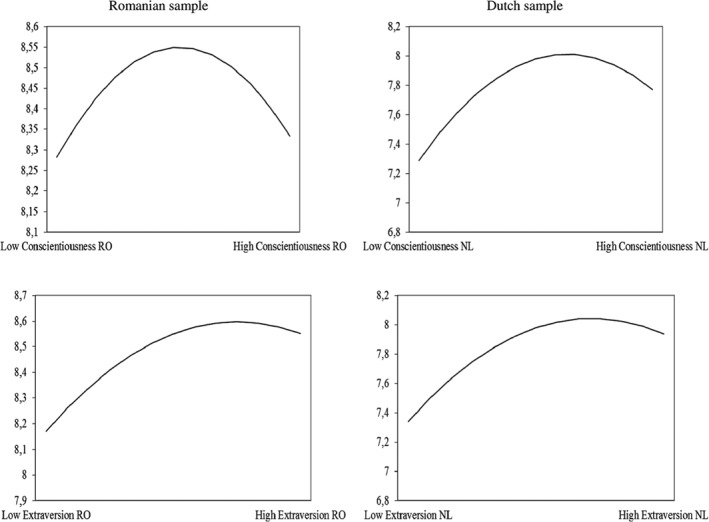
Quadratic relationships between extraversion, conscientiousness and contributions to teamwork in both Romanian and Dutch samples.

Regarding H1, the coefficients for the quadratic term of extraversion were negative and significant in both samples (γ_90_ = −.38, *SE* = .18, *p* < .05 for the Romanian sample, γ_90_ = −.14, *SE* = .04, *p* < .05 for the Dutch sample). Therefore, we can conclude that this hypothesis was supported by the statistical results from both samples. Regarding H2, we have found supporting results only in the case of the Romanian sample (γ_110_ = −.41, *SE* = .18, *p* < .05), but not for the Dutch sample (γ_110_ = −.04, *SE* = .06, *p* > .05). Finally, the hypothesis that anticipated an inverted U‐shaped relationship between conscientiousness and effective contributions to teamwork was supported by results from both samples (γ_120_ = −.32, *SE* = .11, *p* < .05 for the Romanian sample and γ_120_ = −.15, *SE* = .04, *p* < .05 for the Dutch sample). Figure 1 depicts (for both samples) the non‐linear association between extraversion and conscientiousness on the one hand and contributions to teamwork on the other hand.

**Figure 2:**
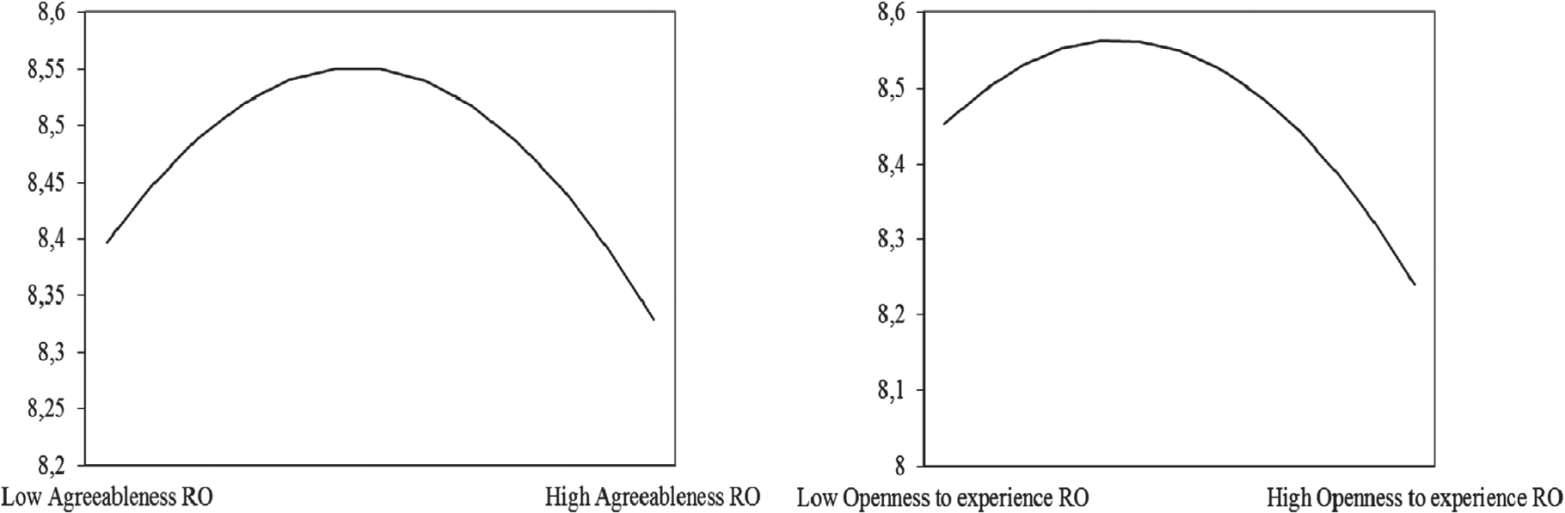
Quadratic relationships for the additional findings in the Romanian sample (agreeableness and openness to experience).

In addition, an emergent finding was that the squared term for openness to experience was also negative and significant in the Romanian sample (γ_100_ = −.41, *SE* = .20, *p* < .05); however, this finding was not replicated in the Dutch sample (γ_100_ = .01, *SE* = .05, *p* > .05). In both samples, neuroticism had no significant quadratic relationship with effective contributions to teamwork (γ_80_ = −.07, *SE* = .12, *p* > .05 for the Romanian sample and γ_80_ = .01, *SE* = .02, *p* > .05). Figure 2 depicts the non‐linear association between openness to experience and agreeableness on the one hand and contributions to teamwork on the other hand for the Romanian sample.

## DISCUSSION

As teamwork becomes increasingly important in organisations, predicting who has effective teamwork skills is of utmost importance in personnel selection. We set out to investigate the “too much of a good thing” effect (Grant & Schwartz, 2011; Pierce & Aguinis, 2013) of personality on contributions to teamwork. We therefore extend the research on the association between personality dimensions and teamwork skills by showing that extraversion and conscientiousness are curvilinearly related to contributions to teamwork. An important strength of the present study is that we tested our hypotheses in two samples from different cultural contexts, and the non‐linear association between conscientiousness and extraversion, on the one hand, and peer‐rated contributions to teamwork was replicated in both samples, while agreeableness and openness to experience displayed a non‐linear associated with contributions to teamwork in one of the samples only.

Extraversion and agreeableness are interpersonal traits and were extensively explored in previous studies addressing the personality‐group outcomes relationships. Our study goes beyond the linearity of their association with group outcomes and shows that the relationship between extraversion and contributions to teamwork is decreasingly positive (as the main effect of extraversion remains positive and significant after adding the squared terms in the equation). Conscientiousness was also extensively explored in previous research as it is closely associated with task performance in groups and our study points towards an inverted U‐shaped association of conscientiousness with contributions to teamwork. We support therefore the existence of a “too much of a good thing” for conscientiousness and extraversion. Moreover, in line with previous research (Peeters et al., 2006) our results show no association between neuroticism and contributions to teamwork.

Our emergent findings suggest that openness to experience has an inverted U‐shaped association with contributions to teamwork. Nevertheless, the fact that this particular pattern was not replicated in the Dutch sample shows that this particular result is not particularly robust. One possible explanation is that the groups in the Romanian sample in which the non‐linear association of openness to experience with contributions to teamwork was found, had to perform a creative task. Groups had a deadline to reach a decision on how to solve the creative task and because group members with extremely high scores on openness to experience often seek new and unconventional experiences, they may disturb the social harmony within the group, increase the likelihood of conflict and ultimately delay the consensus (O'Neill & Allen, 2011; Van Vianen & De Dreu, 2001). Future research, however, could further explore the association between openness to experiences and other creative tasks.

Our conceptualization of the fit between personality and effective contributions to teamwork along with our results has implications for theory and research linking personality to other outcomes important for individuals and organisations by suggesting that it would be a worthwhile endeavour to formulate and test models that specify curvilinear effects of personality traits on such outcomes. Research on disruptive effects of dark sides of personality (Kaiser, LeBreton, & Hogan, 2013) argued that the dark personality traits could reflect extreme values of the five‐factor model dimensions. Our results are in line with this argument and show that, at least for contributions to teamwork, too much or too little extraversion, agreeableness and conscientiousness are detrimental. Future research is needed to develop scales to evaluate the maladaptive (dark personality traits) that possibly explain the results reported in our study.

Finally, relevant to the interpersonal domain, for example, linear associations of interpersonal personality traits—agreeableness and conscientiousness—and citizenship behaviours are well‐documented (see Ilies, Fulmer, Spitzmuller, & Johnson, 2009); perhaps time has come to question the linearity assumptions behind these findings and examine whether employees with some moderate (optimal) levels of these traits are those who perform most citizenship behaviours at work. More generally, we also hope that our work reported herein will stimulate interesting and important research on the complex links between individuals' characteristics and their feelings, functioning and behaviour at work.

### Theoretical and practical implications

Both replicated and contextual results presented in this study are valuable contributions for an applied context. On the one hand, the contextual results obtained for openness to experience create venues for future research. Thus, a relevant research direction for future research would be to identify contingencies that influence the nature of the relationship between openness to experience and contributions to teamwork. On the other hand, the replicated results indicate that there are robust curvilinear patterns, at least in the case of extraversion, agreeableness and conscientiousness. These results contribute to the debate on the validity of personality dimensions in predicting work‐related variables (Le et al., 2011; Rothstein & Goffin, 2006). It is highly relevant to adapt the personnel selection strategies in line with these insights (Converse & Oswald, 2014). For example, when using personality dimensions in personnel selection for jobs involving teamwork, it is relevant to use cut‐off points as high scores on extraversion, agreeableness, openness to experience and conscientiousness are likely to be associated with lower abilities to work together in a team and to collaborate effectively. As argued by Le et al. (2011), discounting applicants with extremely high scores on these personality dimensions might have significant benefits in personnel selection. Such an applied recommendation is particularly supported by the magnitude of the effect sizes obtained for the non‐linear predictors in this study.

### Limitations

Next to its contributions, our study has also several limitations. First, our evaluation of the five personality dimensions was based on a dominance model (average scores of the items included in a scale), while recent research (Carter et al., 2014) argued that non‐linear associations of personality with work‐related variables are better captured under the ideal point measurement models. Due to the fact that our dependent variables are evaluated using two items, and the evaluation of personality in the Dutch sample is also based on two‐item measures, we could not carry out the item response approach suggested by Carter, Dalal, Guan, LoPilato, and Withrow (2017) to detect non‐linear relations. Future research could use the item response approach and explore the extent to which our findings hold under the ideal point measurement models of personality traits. Second, our study used contributions to teamwork as evaluated by peers as a proxy for one's teamwork skills. Future research could directly evaluate teamwork skills in a multidimensional fashion, either by coding the video recordings of team interactions (Annett, Cunningham, & Mathias‐Jones, 2000) or by using behaviorally anchored rating scales (Ohland et al., 2012). Moreover, in our analysis, we used the global scores for the big five dimensions, yet a more specific evaluation of maladaptive traits associated with these dimensions could yield further insights into the relationship between personality and teamwork skills. These more fine‐grained evaluations of teamwork skills and personality could allow for a more specific exploration of the plausible non‐linear associations between (specific) personality trait and various teamwork skills. Finally, the team tasks used in the two studies might reduce the generalizability of our findings and future research could explore whether the non‐linear associations hold in different team contexts.

## CONCLUSIONS

We extend previous research on the non‐linear association between personality and job‐related outcomes by testing (simultaneously) the non‐linear association between the big five dimensions and contributions to teamwork. Our results have important implications for the research exploring the relationship between personality and engagement in teamwork as we show that extraversion and conscientiousness are non‐linearly associated with one's contribution to teamwork. In practical terms, our results emphasise the need to adapt personnel selection and recruiting principles in order to account for the non‐linearity of the relationships between personality dimensions and teamwork skills.
